# The functional and inflammatory response of brain endothelial cells to Toll-Like Receptor agonists

**DOI:** 10.1038/s41598-018-28518-3

**Published:** 2018-07-04

**Authors:** Rebecca H. Johnson, Dan T. Kho, Simon J. O’ Carroll, Catherine E. Angel, E. Scott Graham

**Affiliations:** 1 Centre for Brain Research, Auckland, New Zealand; 2 Department of Pharmacology and Clinical Pharmacology, Auckland, New Zealand; 3 Department of Anatomy and Medical Imaging, School of Medical Sciences, Faculty of Medical and Health Sciences, University of Auckland, Auckland, New Zealand; 4 School of Biological Sciences, Faculty of Science, University of Auckland, Auckland, New Zealand

## Abstract

Toll-Like receptors (TLRs) represent an important early warning mechanism for the immune system to detect infection or tissue damage. The focus of this research was to determine the neuroinflammatory responses to commercial TLR ligands and their effects on brain endothelial barrier strength. Using biosensor technology we screened TLR ligands to all human TLRs and found that the brain endothelial hCMVECs cell line only responded to Poly(I:C) (TLR3-ligand), LPS (TLR4-ligand) and Imiquimod (TLR7 ligand). Both Poly(I:C) and LPS induced pronounced pro-inflammatory cytokine secretion as expected, whereas Imiquimod did not induce secretion of any pro-inflammatory cytokines. Using ECIS technology to measure endothelial barrier function, LPS and Poly(I:C) both acutely reduced barrier-strength, whereas Imiquimod caused immediate and sustained strengthening of the barrier. Further cytokine and ECIS studies showed that Imiquimod could abrogate some of the pro-inflammatory responses to Poly(I:C) and LPS. Most surprisingly, PCR revealed that the hCMVECs lacked TLR7 but expressed both TLR3 and TLR4 and did not respond to other structurally different TLR7 ligands. These data demonstrate that brain endothelial cells can be regulated by TLR 3 and TLR4 ligands in a pro-inflammatory manner and have receptors to Imiquimod, distinct to the classical TLR7, that function in an anti-inflammatory manner.

## Introduction

Endothelial cells of the blood-brain barrier (BBB) are an integral component in the regulation of immune cell entry into the central nervous system (CNS). The BBB is composed of a monolayer of these specialised brain (cerebrovascular) endothelial cells (BECs) whose properties ultimately maintain cerebral homeostasis^1,2^. The BECs differ from those of the periphery due to a specific and selective transport barrier, a metabolic barrier, and a physical barrier comprised of a high density of tight and adherens junctions, all of which ensure a high resistance to molecules, polar solutes and ions^1^.

The Toll-Like Receptor (TLR) family, discovered in the late 1990s are innate immune pattern recognition receptors^3^. The primary function of these receptors is the recognition of specific and distinct conserved endogenous and exogenous molecular patterns (Danger Associated Molecular Patterns (DAMP) and Pathogen Associated Molecular Patterns (PAMP) respectively)^4,5^. These receptors mediate their effects through the TIR-domain-containing adaptor-inducing interferon-β (TRIF) or myeloid differentiation primary response gene 88 (MyD88) pathways^6,7^. Once activated these receptor-dependent pathways then mediate the release of cytokines, chemokines and anti-microbial peptides^8^. TLR expression and function has been extensively studied in endothelial cells of the peripheral immune system and has been found in pulmonary, dermal, human umbilical vein, intestinal and coronary artery endothelial cells^4,9,10^. Research by Nagyoszi *et al*., into the expression of TLRs in endothelial cells of the BBB has reported the expression of TLR 2, 3, 4 and 6 in the hCMEC/D3 line^11^. In the same cell line, Li *et al*., reported the expression of these and TLR1^12^. As the majority of research focuses on the CNS response to TLR activation it is the pro-inflammatory response of TLR2 and 4, traditionally bacterial TLRs (in that they recognise bacterial triacylated/diacylated peptides or lipopolysaccharide respectively) that have been the most extensively studied^13–16^.

We hypothesized that brain endothelial cells would express the TLRs noted above which would respond to their cognate ligands in the classical TLR-mediated pro-inflammatory manner. Initially, xCELLigence RTCA biosensor technology^17,18^ was used to determine the global responsiveness of human cerebral microvascular endothelial cells (hCMVECs) to a panel of commercial TLR ligands. This revealed marked responses to the TLR3 ligand Poly(I:C) and TLR4 ligand lipopolysaccharide (LPS) and to the TLR7 ligand Imiquimod, but not to any of the other TLR ligands. Inflammatory cytokine analysis revealed that Poly(I:C) and LPS were highly pro-inflammatory, whereas Imiquimod did not induce the secretion of any pro-inflammatory cytokines measured. Endothelial barrier function was measured using Electric-cell Substrate Impedance Sensing (ECIS) technology, considered the most sophisticated technology for *in vitro* barrier measurements^19–21^. ECIS demonstrated that Poly(I:C) and LPS both caused an acute reduction in barrier strength similar to our previous observations with TNFα and IL-1β^22^, whereas Imiquimod caused an immediate and sustained strengthening of the barrier. PCR analysis revealed that the brain endothelial cells only expressed TLR3 and TLR4 mRNA, and most surprisingly lacked TLR7 meaning the observed effects were independent of TLR7. Further studies revealed that Imiquimod could abrogate some of the pro-inflammatory responses induced by Poly(I:C) and LPS.

Imiquimod is FDA approved as a topical cream for the treatment of superficial basal cell carcinoma, external genital warts and actinic keratinosis^23–25^, where its mechanism of action is to orchestrate a pro-inflammatory response through TLR7. The data presented here are the very first demonstration of Imiquimod mediating a barrier enhancing and immunosuppressive effect on human BECs via a non-TLR7 receptor. These data also reveal the power of biosensor technologies to uncover the cellular responsiveness to ligands, irrespective of knowledge pertaining to the presence or absence of the ligand’s target receptors. More importantly with regards to neuroinflammation, these data indicate that brain endothelial cells have anti-inflammatory mechanisms to protect themselves against the pro-inflammatory milieu that may occur during peripheral or neuroinflammatory events.

## Results

### Pharmacological responsiveness of hCMVECs to viral TLR ligands

**Figure 1:**
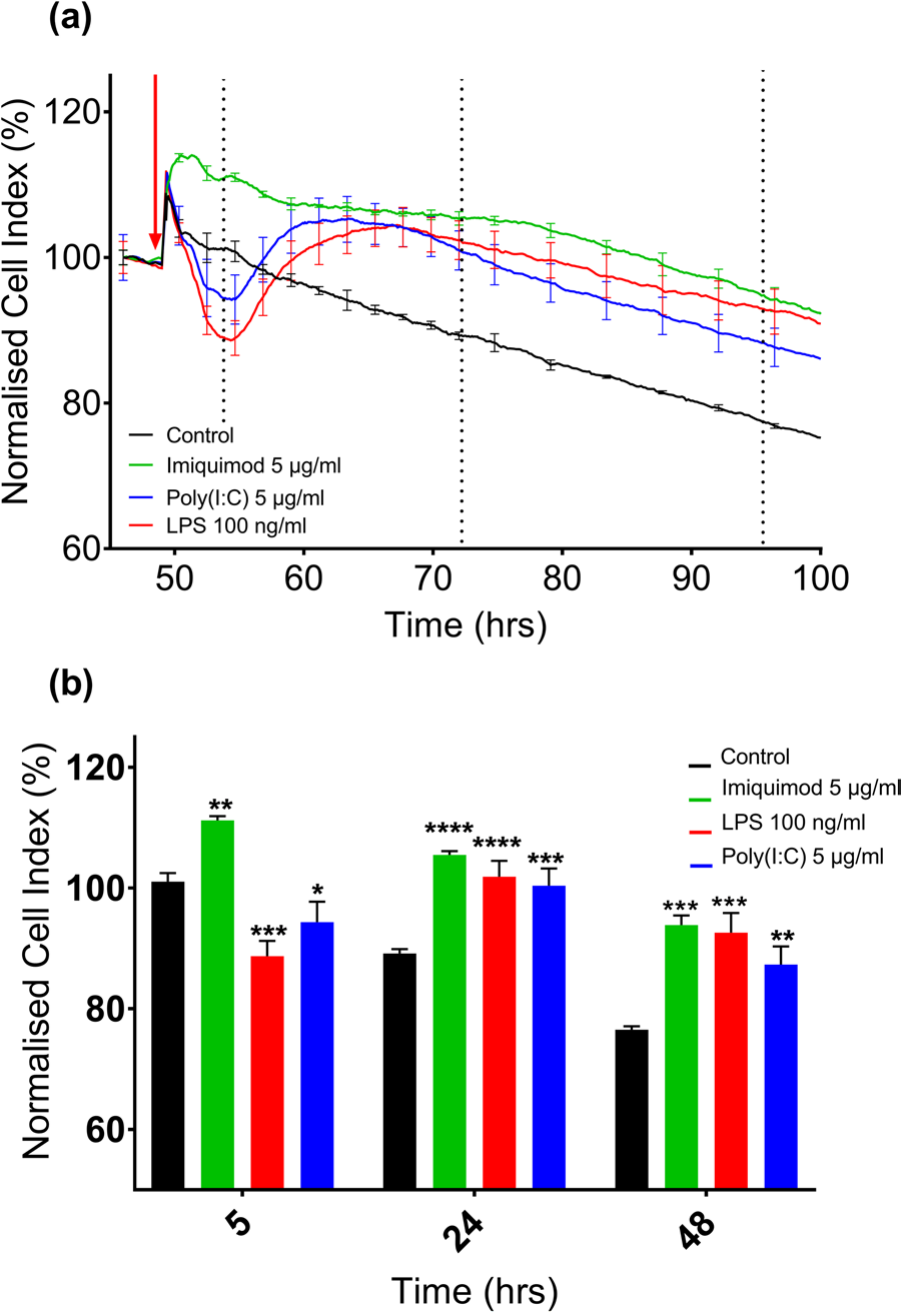
Analysis of hCMVEC responsiveness to TLR ligands measured using xCELLigence RTCA Biosensor technology. (**a**) TLR agonists were administered to confluent hCMVECs on an xCELLigence 96 well plate with Poly(I:C) 5 µg/mL (blue), Imiquimod 5 µg/mL (green), LPS 100 ng/ml (red), or media-only control (black). Data shown has been normalized (100%) prior to the addition of ligands (raw data shown in Supplemental Fig. 2). Curves represent the mean ± SD of three individual wells with a red arrow indicating ligand addition and dashed lines indicating 5, 24 and 48 hours following ligand addition. (**b**) Cell Index of hCMVEC 5, 24 or 48 hours following addition of Poly(I:C), LPS, Imiquimod or media-only control. Significance compared to control unless otherwise stated; *p < 0.05, **p < 0.01, ***p < 0.001, ****p < 0.0001.

To investigate the pharmacological responsiveness of hCMVECs to the TLR ligands, we used the xCELLigence RTCA biosensor technology. xCELLigence is able to show cellular responsiveness in a temporal manner, measured using the arbitrary unit ‘Cell Index’ (CI)^17,18,22,26^. The ligands of TLR1/2 (Pam3CSK), TLR2/6 (Pam2CSK), TLR 8 (R-848), and TLR9 (CpG-ODN 2006), did not mediate a change in hCMVEC CI compared to that of the vehicle treated control (Supplemental Fig. 1). In contrast to this, a range of concentrations of Poly(I:C), the synthetic TLR3 ligand and LPS the TLR4 ligand, mediated an observable acute decrease in CI compared to control (Fig. 1). Following this decrease, the CI then significantly increased above control, a pattern which has been previously observed with the pro-inflammatory cytokines TNFα and IL-1β and is thought to be indicative of a pro-inflammatory response^22^. In contrast to this, 5 µg/mL of Imiquimod exerted an immediate and sustained significant increase in CI compared to control. This data therefore indicates that hCMVECs are responsive to this small Imidazoquinoline (Fig. 1), but in a manner that differs to the response to Poly(I:C) and LPS.

### Effect of Poly(I:C), LPS, and Imiquimod on hCMVEC cytokine, chemokine and cell adhesion molecule secretion

**Figure 2:**
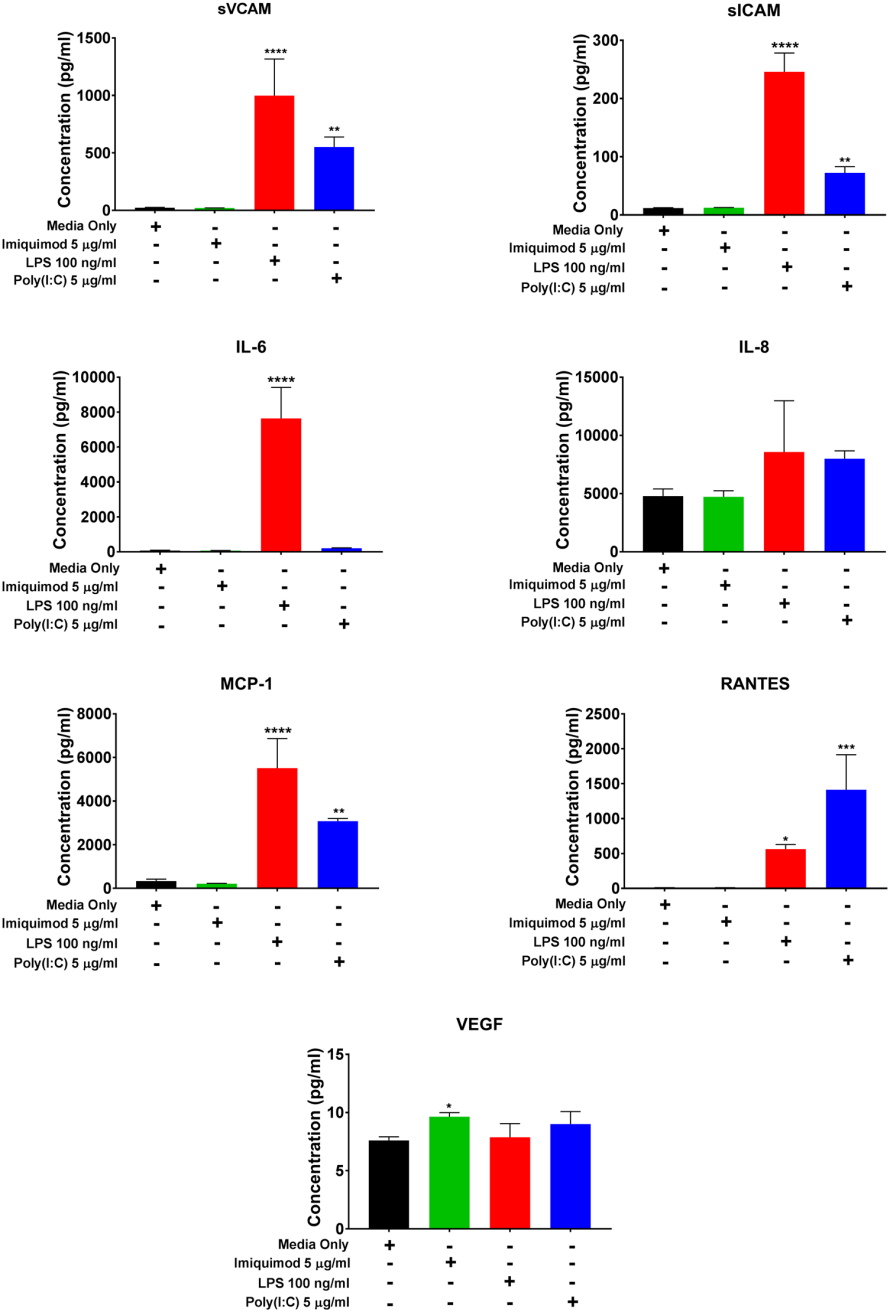
Characterization of hCMVEC secretion following administration of Imiquimod (5 µg/mL), LPS (100 ng/ml), Poly(I:C) (5 µg/mL), or media-only control at 48 hours. The concentration of soluble factors in the conditioned media was measured using multiplexed cytometric bead array. Cultured media was collected 72 hours following treatment and assayed using a cytokine panel chosen based on previous research^17^. Data shows the mean ± SD (n = 3). Significance compared to control (unless otherwise stated); *p < 0.05, **p < 0.01, ***p < 0.001, ****p < 0.0001.

Our previous research has shown that activated hCMVECs secrete a number of pro-inflammatory mediators including IL-8 (CXCL8), IL-6, monocyte chemoattractant protein-1 (MCP-1/CCL-2), RANTES (CCL5), soluble ICAM-1 (sICAM-1/sCD54) and soluble VCAM-1 (sVCAM-1/sCD106) upon activation by IL-1β and TNFα^22^. We therefore measured the presence of these cytokines, chemokines and soluble cell adhesion molecules using Cytometric Bead Array to determine whether Poly(I:C), LPS or Imiquimod induce a similar pro-inflammatory response (Fig. 2). Under basal conditions, the concentration of the majority of the pro-inflammatory panel was very low or undetectable, as observed previously^22^. Treatment with Imiquimod (5 µg/mL) did not induce the secretion of any of the pro-inflammatory cytokines. In contrast to this, treatment of the hCMVECs with LPS (100 ng/ml) mediated a substantial increase in all the pro-inflammatory cytokines, chemokines and soluble cell adhesion molecules but did not mediate a substantial increase in VEGF (Fig. 2). Poly(I:C) (5 µg/mL) also induced the production of most of the panel of pro-inflammatory cytokines, however RANTES was the only factor induced to a greater extent by Poly(IC) in comparison to LPS. Thus, it seems that whilst the hCMVECs are responsive as expected to Poly(I:C) and LPS, their responses differ, especially with regards to the chemokine RANTES. In addition to this, hCMVECs did not respond to Imiquimod in a classical pro-inflammatory manner. Although Imiquimod increases VEGF significantly, the effect size is very small as the VEGF concentration measured in these cultures was very low (≤10 pg/mL) None of the other TLR agonists had any significant influence on VEGF at this time point (Fig. 2).

### Effect of Poly(I:C), LPS, and Imiquimod on brain endothelial barrier strength

**Figure 3:**
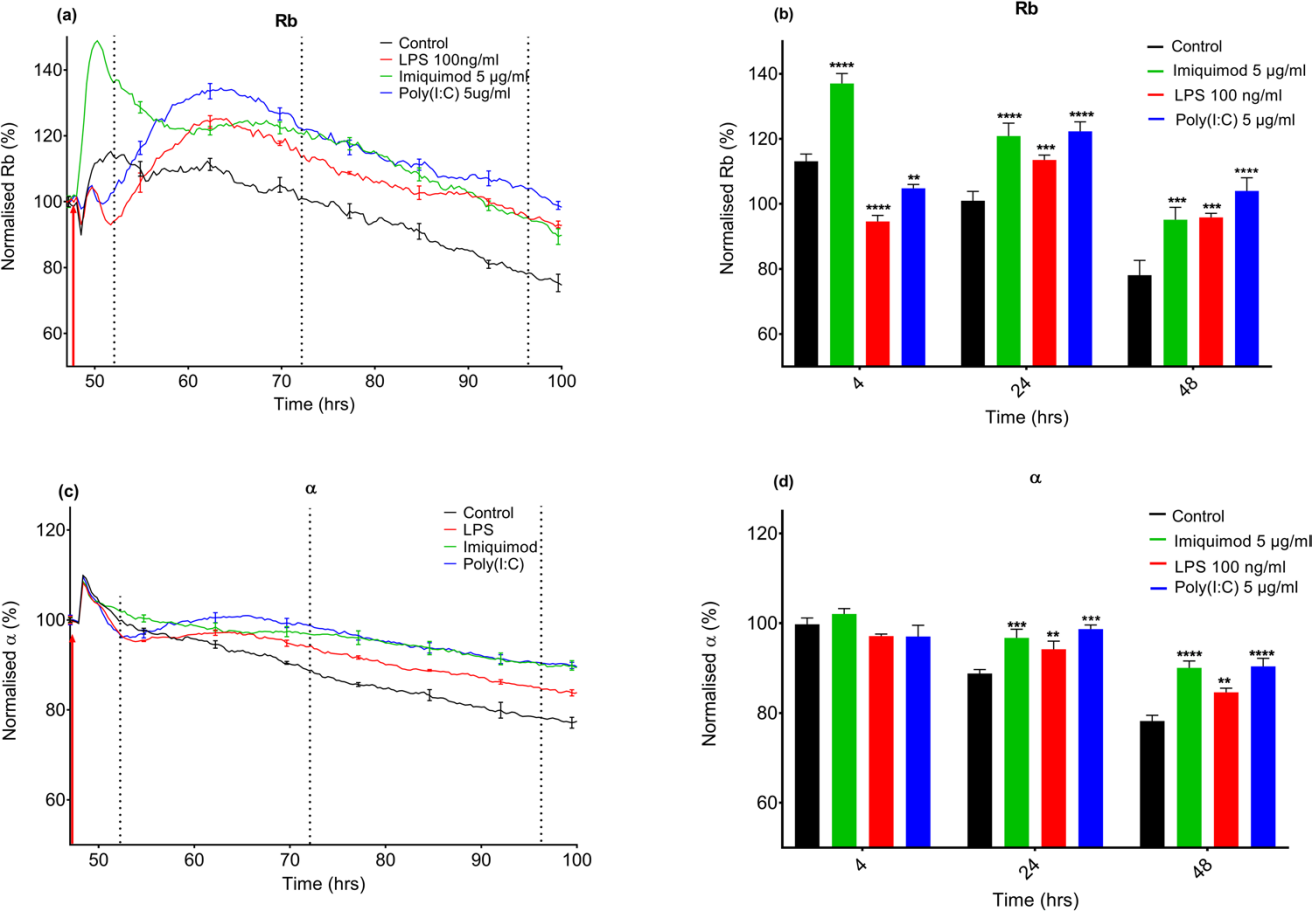
Modelling of hCMVEC adhesion following Poly(I:C), LPS and Imiquimod treatment. TLR agonists Imiquimod (5 µg/mL) LPS (100 ng/ml), Poly(I:C) (5 µg/mL), or media-only control were administered to confluent hCMVECs 48 hours post seeding (represented by a red arrow) on an ECIS 96W20idf plate. Data shown has been normalized (100%) prior to the addition of ligands (raw data shown in Supplemental Fig. 3). Multi-frequency measurements were taken and this data was modelled using ECIS Software V.1.2.163.0 PC. (**a**) Shows the modelled intercellular adhesion (Rb) whilst (**b**) shows Rb 4, 24 and 48 hours post-treatment. (**c**) Shows the modelled basolateral adhesion (α). (**d**) Shows α at 4, 24, and 48 hours post-treatment for statistical comparisons. The modelled data is from a single experiment where each curve represents the mean ± SD of three individual wells. Representative of at least three independent experiments. Significance compared to control (unless otherwise stated). *p < 0.05, **p < 0.01, ***p < 0.001, ****p < 0.0001.

xCELLigence RTCA Biosensor technology tells us the overall impedance (converted into Cell Index) of the hCMVEC monolayer and thus overall endothelial barrier integrity; using ECIS Zθ technology, multi-frequency impedance measurements can be broken down into the components of cellular basolateral adhesion (focal adhesion strength; α) and paracellular adhesion (determined by the expression of junctional molecules and cell-cell contact surface area; Rb)^19,21^. We therefore used ECIS to determine which aspect of barrier function was affected by the TLR ligands (Fig. 3). Interestingly, it was the paracellular barrier strength (Rb) that was most affected by LPS, Poly(I:C) and Imiquimod. On close inspection of the ECIS data for Poly(I:C) and LPS, it can be seen that both cause an initial reduction in α for 4–6 hours, whereas whilst there is a reduction in Rb, it is for a much shorter period of time. Following this both Poly(I:C) and LPS induce a marked increase in Rb and α which is sustained for the remainder of the experiment. In contrast to this, Imiquimod significantly increased the intercellular barrier strength (Rb) immediately, which was sustained for at least 50 hours (Fig. 3a). Interestingly, α is not seen to increase until 12 hours following treatment, indicating that whilst Imiquimod does have an effect on basolateral adhesion, it is delayed compared to the Rb. This further demonstrates the differing effects of these TLR ligands on the hCMVEC barrier function.

### TLR7 expression and activation

TLR7 activation by its appropriate ligand traditionally invokes a pro-inflammatory anti-viral response, and this was therefore the expected response to the TLR7 ligand Imiquimod^27^. As the observed cytokine response profile of Imiquimod was not inherently pro-inflammatory, it was hypothesized that it is not activating TLR7 in these brain endothelial cells. Previous research by Nagyoszi et. al., and Li *et al*., did not find any evidence of TLR7 in the hCMEC/D3 brain endothelial cell line; however the functional effect of Imiquimod was not measured in these experiments^11,12^.

**Figure 4:**
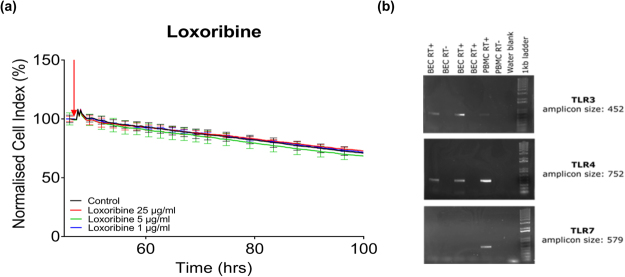
Analysis of hCMVEC responsiveness to TLR7 ligand Loxoribine and PCR of TLR 3, 7 and 4. (**a**) The TLR7 agonist Loxoribine 25 µg/mL (red) 5 µg/mL, (green), 1 µg/ml (blue), and untreated media-only control (black) was administered to confluent hCMVECs 48 hours post-seeding on an xCELLigence 96 well plate, indicated by the red arrow. Data shown has been normalized (100%) prior to the addition of ligands (raw data shown in Supplemental Fig. 5). Curves represent the mean ± SD of three individual wells, (normalised prior to addition of ligands) and are representative of at least three independent experiments. (**b**) PCR of untreated hCMVEC cDNA for TLR 3, 7 and 4. RT+ indicates the presence of reverse transcriptase. cDNA from human PBMC was used as the positive control. PCR products were separated by agarose gel electrophoresis and visualized using RedSafe. A 100 bp DNA ladder was used for amplicon sizing. Images are representative of at least three independent experiments.

To determine whether the brain endothelial cells were similarly responsive to alternative TLR7 agonists, a number of commercially available TLR7 agonists (Gardiquimod (Inivogen, Cat#: tlrl-gdqs), Loxorobine (Invivogen, Cat#: tlrl-lox), CL097 (Invivogen, Cat#: tlrl-c97-5) and CL307 (Invivogen, Cat#: tlrl-c307)), each with differing levels of specificity/receptor pharmacology were screened using the xCELLigence RTCA biosensor. None of the ligands were found to mediate a change in the temporal CI of the hCMVECs (Fig. 4a and Supplemental Fig. 4). As this was in stark contrast to the results of Imiquimod treatment, PCR was undertaken to determine whether TLR7 mRNA was present within the hCMVECs (Fig. 4b). Whilst TLR3 and TLR 4 mRNA were observed, there was no detectable TLR 1, 2, 6, 7, 8 or 9 mRNA in the hCMVECs, indicating that the response to Imiquimod was clearly independent of TLR7 and that the TLR mRNA pool in this brain endothelial cell line differs to the hCMEC/D3 cell line^11,12^. These results clearly indicate that Imiquimod is activating an alternative receptor on these BECs, highlighting the importance of pharmacological assessment, as performed in this study using the xCELLigence Biosensor.

### Effect of co-treatment of TLR ligands on barrier strength

**Figure 5:**
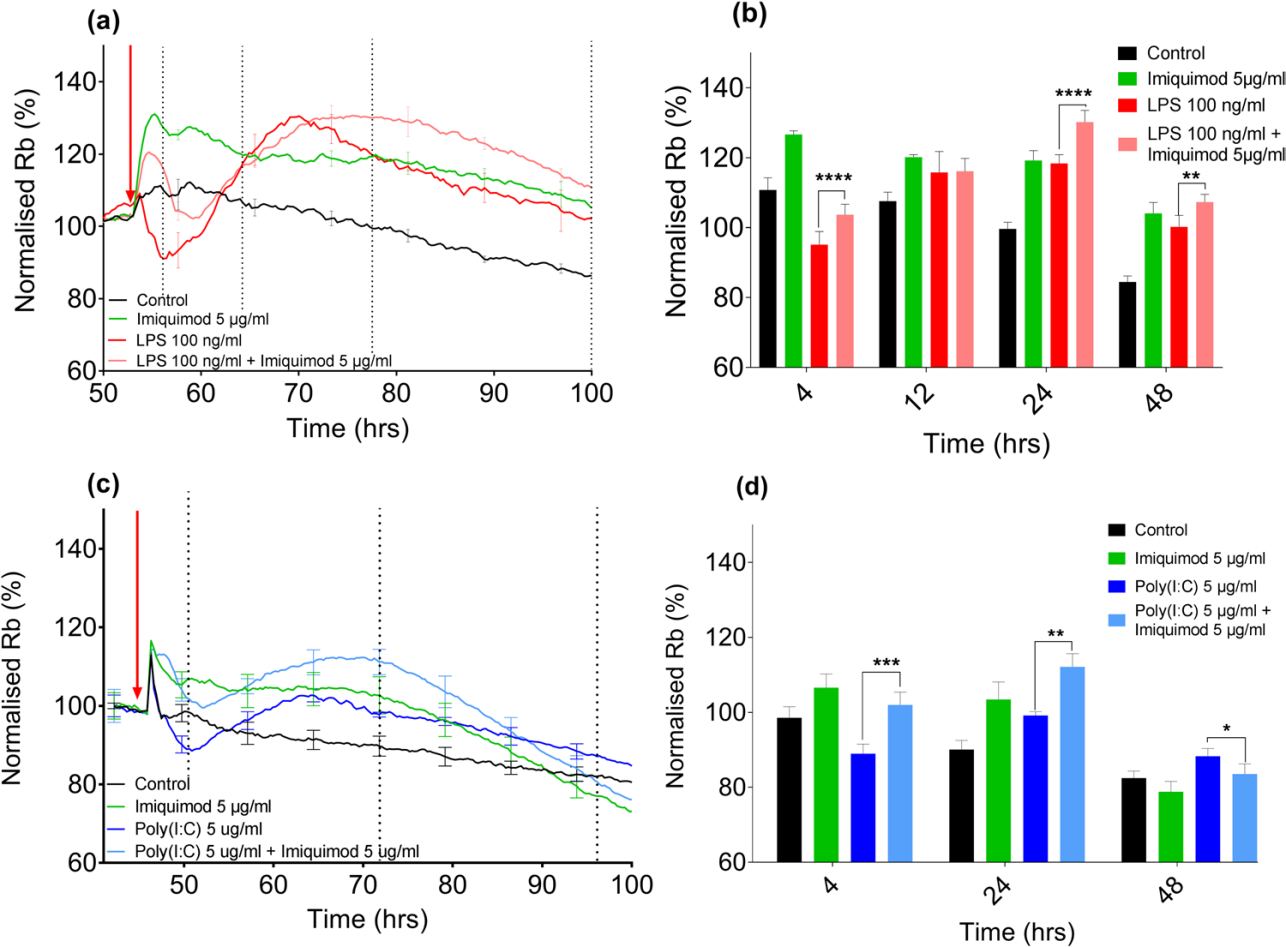
Modelled Rb of hCMVEC adhesion. TLR agonists were administered to confluent hCMVECs 48 hours post-seeding (represented by a red arrow) on an ECIS 96W20idf plate with (**a**) LPS (100 ng/ml), Imiquimod (5 µg/mL), LPS (100 ng/ml) plus Imiquimod (5 µg/mL), or untreated media-only control. (**b**) Shows the LPS treatments above 4, 12, 24 and 48 hours post-treatment. (**c**) Poly(I:C) (5 µg/mL), Imiquimod (5 µg/mL), Poly(I:C) (5 µg/mL) plus Imiquimod (5 µg/mL), and untreated media-only control. (**d**) Shows the Poly(I:C) treatments above 4, 24, and 48 hours post-treatment. Multi-frequency measurements were taken and this data was later modelled using ECIS Software V.1.2.163.0 PC. Data shown has been normalized (100%) prior to the addition of ligands (raw data shown in Supplemental Fig. 4). The modelled data is from a single experiment where each curve represents the mean ± SEM of three individual wells. Representative of at least three independent experiments. Significance; *p < 0.05, **p < 0.01, ***p < 0.001, ****p < 0.0001.

The novel finding that Imiquimod is able to mediate strengthening of the brain endothelial intercellular barrier in a TLR7 independent manner and did not directly induce secretion of pro-inflammatory cytokines led us to hypothesize that co-administration of this ligand may attenuate the pro-inflammatory cytokine and chemokine release, and barrier effects of LPS or Poly(I:C). In co-administration ECIS experiments, when the endothelial cells were treated with both Imiquimod and LPS the initial acute weakening of the barrier was altered and an acute increase, (similar to that displayed following Imiquimod treatment), was observed followed by an acute decrease (normalized data Fig. 5, non-normalized Supplemental Fig. 3). The LPS and Imiquimod co-treated cells then had a marked increase in Rb greater than that observed for either LPS alone or Imiquimod alone. This increase in barrier strength Rb was also sustained for longer (Fig. 5a).

When cells were treated with both Poly(I:C) and Imiquimod, an overall increase in Rb was observed for the entire time-course, compared to the Poly(I:C) treatment alone, indicating that the barrier was stronger than the control treated cells for the entire period. The co-treatment had the same temporal pattern as that of cells treated with Poly(I:C) alone but the barrier was strengthened by an extra ~10% above the Poly(I:C) alone values. The influence of Imiquimod does not simply look additive to Poly(I:C), and the temporal changes induced by Imiquimod treatment are different for LPS compared to Poly(I:C) (Fig. 5c,d).

### Effect of co-treatment of TLR ligands on hCMVEC cytokine, chemokine and cell adhesion molecule secretion

**Figure 6:**
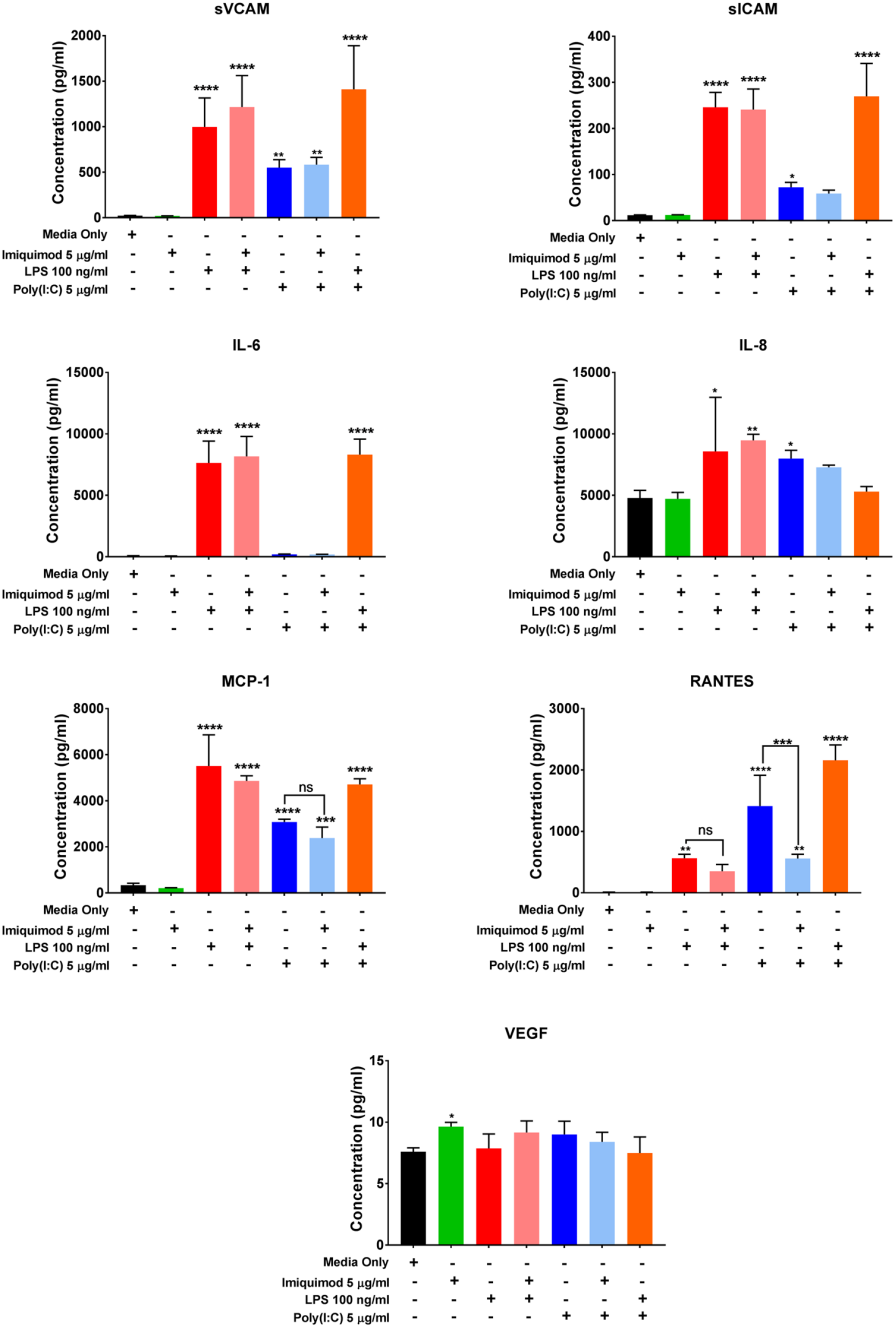
Characterization of hCMVEC secretion following administration of Poly(I:C) (5 µg/mL), Imiquimod (5 µg/mL), LPS (100 ng/ml), LPS (100 ng/ml) and Imiquimod (5 µg/mL), Poly(I:C) (5 µg/mL) and Imiquimod (5 µg/mL), and Poly(I:C) (5 µg/mL) and LPS (100 ng/ml) or untreated media-only control at 24 hours post-treatment. The concentration of soluble factors in the conditioned media was measured using multiplexed cytometric bead array. Cultured media was collected at 24 hours following treatment and assayed using a cytokine panel chosen based on previous research^17^. Data shows the mean ± SD (n = 3). Significance is compared to control unless otherwise indicated by the horizontal linking bars. *p < 0.05, **p < 0.01, ***p < 0.001, ****p < 0.0001.

As Imiquimod was able to alter the Poly(I:C) and LPS mediated effects on paracellular barrier strength, it was hypothesized that Imiquimod would also alter the secretion of pro-inflammatory cytokines, chemokines and cell adhesion molecules. Cytometric bead array was used to measure the soluble factors accumulated in the media 24 hours post-treatment (Fig. 6). Interestingly, Imiquimod had differing effects on the pro-inflammatory factors induced by Poly(I:C) or LPS treatment. Treatment with Imiquimod and Poly(I:C) resulted in a significant decrease in the production of RANTES compared to Poly(I:C) alone. However the production of sICAM-1, sVCAM- 1, IL-6, IL-8, MCP-1 and VEGF were unaffected at this time-point. Imiquimod did not significantly affect the secretion of any of the cytokines induced by LPS over the 24 h period (Fig. 6).

**Figure 7:**
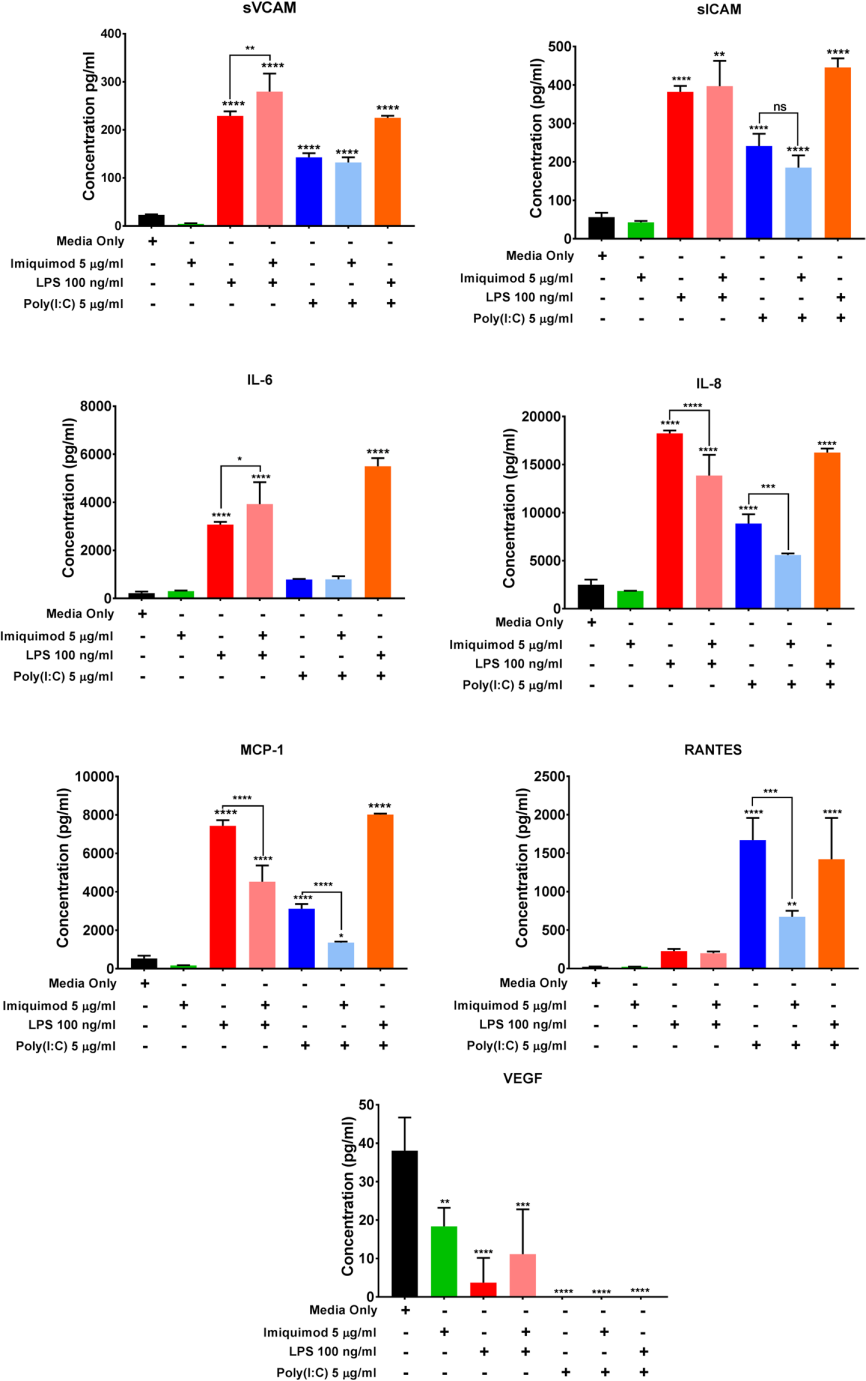
Characterization of hCMVEC secretion 72 hours following administration of Poly(I:C) (5 µg/mL), Imiquimod (5 µg/mL), LPS (100 ng/ml), LPS (100 ng/ml) and Imiquimod (5 µg/mL), Poly(I:C) (5 µg/mL) and Imiquimod (5 µg/mL), and Poly(I:C) (5 µg/mL) and LPS (100 ng/ml) or untreated media-only control post-treatment. The concentration of soluble factors in the conditioned media was measured using multiplexed cytometric bead array. Cultured media was collected at 72 hours following treatment and assayed using a cytokine panel chosen based on previous research^17^. Data shows the mean ± SD (n = 3). Significance is compared to control unless otherwise indicated by the horizontal linking bars. *p < 0.05, **p < 0.01, ***p < 0.001, ****p < 0.0001.

When soluble factors were allowed to accumulate for 72 hours post-treatment (Fig. 7), further trends became apparent. In the Poly(I:C) and Imiquimod co-treatment, the concentration of RANTES, IL-8 and MCP-1 were significantly reduced in comparison to the Poly(I:C) alone. In the co-treatment of Imiquimod with LPS, the concentration of IL-8 and MCP-1 were both significantly reduced with the inclusion of Imiquimod. In contrast VCAM and IL-6 concentrations were slightly greater in the co-treated cells. These data show that Imiquimod can influence the factors secreted following LPS and Poly(I:C) activation of the cells and that the effects differ with respect to the activating TLR ligand. VEGF secretion was reduced by Imiquimod, LPS and Poly(I:C) at 72 h, but we stress that the concentrations of VEGF being produced by these cells is very low (Fig. 7). One mechanism by which Imiquimod may be attenuating the production of pro-inflammatory chemokines is the secretion of ‘anti-inflammatory’ cytokines (e.g. IL-10, IL-4, IL-5, IL-7 and IL-13). A number of these have been found to inhibit the production of IL-6, IL-8, MCP-1, and RANTES. However, none of these anti-inflammatory cytokines were detected in the cultured media across an extended 72 hour collection period from cells concurrently or singly treated with the TLR ligands, indicating either that they are not produced under these conditions, or not produced by the cells at all (data not shown).

## Discussion

The goal of the study was to determine the functional response of BECs to TLR ligands and to understand how these ligands are able to regulate BECs to advance our understanding of the innate ability to sense these danger signals.

Using the RTCA xCELLigence Biosensor technology we were able to assess the temporal effects of TLR ligands on the BECs, and thus determine the global response of these cells^17^. This technology is an incredibly powerful system for ascertaining the cellular responsiveness of cells to a stimulus. Here the xCELLigence assays were performed independently of knowing whether the classical receptor target was present, which was conducted later by PCR. This strategy revealed that agonists with pharmacology to TLR3, TLR4 and TLR7 activated the BECs. The Biosensor technology also allowed us to quickly and easily ascertain that the hCMVECs were not responsive to various other TLR ligands, a fact later corroborated by PCR confirming that the hCMVECs do not express TLR1, TLR2, TLR6, TLR8 and TLR9 mRNA under basal conditions. The PCR also revealed that the hCMVECs did not express TLR7 which is the pharmacological receptor target for Imiquimod. This implies that all of the functional responses mediated by Imiquimod observed in this study were independent of TLR7.

We have previously found that hCMVECs secrete substantial amounts of pro-inflammatory cytokines, chemokines and cell adhesion molecules following stimulation with TNFα and IL-1β^22^. However the extent to which the cells produce these factors differs by stimulatory cytokine. Cytometric bead array analysis determined that LPS and Poly(I:C) both induced IL-6, IL-8, MCP-1, sVCAM-1, sICAM-1, and RANTES; with LPS inducing all but RANTES to a greater extent than Poly(I:C). The potency of Poly(I:C) to induce the secretion of RANTES has been observed previously in airway epithelial cells, and this chemokine has been found to play a key role in the control of viral infection^28,29^. ECIS is an excellent technology for measuring endothelial barrier function^19,30^ and is the only technology available with the ability to separate out changes in the paracellular barrier and the basolateral focal adhesion strength from overall impedance^21^. The ECIS biosensor has previously been used to determine the effect of CB2R agonists on LPS induced permeability of primary brain endothelial cells^31^. In this study, 50 ng/ml LPS induced a marked decrease in barrier resistance compared to normalized control. However, similarly to the results observed on the hCMVECs, following an acute decrease the resistance of the barrier does start to increase, with measurements ending 36 hours post-treatment. Whilst these primary brain endothelial cells show decreased barrier strength for substantially longer than the hCMVECs, the pattern of recovery and re-strengthening is consistent with the results we have seen following LPS treatment. This pattern is also very similar to that previously observed following TNFα or IL-1β treatment, indicating that this profile may be a characteristic response to pro-inflammatory stimulus^22^. The ability of brain endothelial cells to withstand a significant pro-inflammatory stimulus before recovery is at odds with what is known of systemic endothelial cells, which typically become leaky and cause tissue eodema. In contrast to the acute weakening observed with the pro-inflammatory cytokines^22^ and TLR3/4 agonists, Imiquimod caused a rapid and sustained increase in paracellular barrier strength. This profile is currently unique to Imiquimod, as it has not been observed for any other drug or treatment we have conducted.

The novel observation that Imiquimod is able to increase paracellular barrier strength led us to hypothesize that co-treatment of LPS/Poly(I:C) with Imiquimod would ‘over-ride’ the inflammatory-stimulus induced acute decrease in barrier strength. Imiquimod was indeed able to alter the LPS and Poly(I:C) induced changes in barrier resistance. This demonstrated that when the ligands were given simultaneously, the Imiquimod response dominated over either the LPS or the Poly(I:C) responses with a sustained improvement in barrier strength. In contrast, by 24 hours Imiquimod was only able to significantly attenuate the production of RANTES in the Poly(I:C) treated cells, with further downward trends in MCP-1 and IL-8 not observed until 72 hours post-treatment. This indicates that any effect Imiquimod has on cytokine/chemokine production may be limited to the upstream pathway of these key chemokines. These are very intriguing observations that show the specificity of the Imiquimod response to selective aspects of the pro-inflammatory cascade mediated by both TLR3 and TLR4. RANTES was not induced significantly by LPS, therefore there was no response for Imiquimod to suppress.

The use of Imiquimod in the treatment of superficial basal cell carcinoma and other skin conditions is based on the ability of this molecule to activate TLR7 and thus exert an increase in inflammatory cytokines and type I IFNs^23–25^. Whilst previous research indicates that TLR7 is not expressed in a human brain endothelial cell line, the responsiveness of hCMVEC to Imiquimod led us to question whether TLR7 was expressed by these cells^11^. Treatment of hCMVECs with a large panel of TLR7/8 ligands demonstrated that the cells were unresponsive to these which was further explained by a failure to detect TLR7 mRNA by PCR. Whilst the receptor(s) mediating these effects is at this stage unknown, the response mediated by Imiquimod is highly significant to neuroinflammatory conditions where a reduction in the magnitude of neuroinflammation is sought and where protection of the BBB is required. It is therefore important in future studies to identify the receptor and signaling networks involved in the TLR7-independent anti-inflammatory effects of Imiquimod.

## Materials and Methods

### Materials

TLR ligands used in this study were Pam3CSK (EMC Biochemicals, Cat#: L2000), PAM2CSK (EMC Biochemicals, Cat#: L2020), Poly(I:C) (InivoGen, Cat#: Tlr-pic), Lipopolysaccharide (LPS; Sigma-Aldritch Cat#: L4391), Imiquimod (InvivoGen Cat#: Tlr-imiq), R-848 (IMGENEX, Cat#: IMG-2208), CpG ODN-2006 (InvivoGen, Cat#: Tlr-hodnb), Gardiquimod (Inivogen, Cat#: tlrl-gdqs), Loxorobine (Invivogen, Cat#: tlrl-lox), CL097 (Invivogen, Cat#: tlrl-c97-5) and CL307 (Invivogen, Cat#: tlrl-c307) at concentrations as detailed in the corresponding figure legends. All TLR ligands (with the exception of LPS) were supplied with endotoxin levels <0.001 EU/µg and were made up in sterile endotoxin free water.

### Cell culture

The human cerebral microvascular endothelial cell line (hCMVECs), immortalised with the simian virus 40 large T antigen was purchased from Applied Biological Materials Inc. (Cat#: T0259) and used between passages 6–16 with no observable passage-dependent differences. For standard tissue culture, cells were grown in media composed of M199 medium (Gibco CA, USA) supplemented with 10% fetal bovine serum (FBS) (Sigma), 1 µg/ml hydrocortisone (Sigma), 3 µg/ml human basic fibroblast growth factor (hFGF); Peprotech), 1 µg/ml human epidermal growth factor (hEGF; Peprotech), 10 µg/ml heparin (Sigma), 2 mM Glutamax (Gibco CA, USA) and 80 µM butyl cyclic-AMP (Sigma). Cells were cultured at 37 °C in a dehumidified atmosphere of 5% CO_2_ on Nunc T75 flasks (Falcon, Cat# 156499) that had been coated with 1 µg/cm^2^ rat collagen (Gibco, Cat# A1048301) diluted in 0.02 M glacial acetic acid solution.

### Characterization of pharmacological responsiveness using xCELLigence technology

hCMVEC responsiveness to a range of TLR ligands was analysed using the xCELLigence real time cell analyser (xCELLigence RTCA-SP system bundle, ACEA Biosciences Inc.). Gold electrodes are interdigitated over the base of a 96-well plate, sending a fixed frequency (10 kHz) through the cell monolayer. This allows the measurement of well impedance which is converted to a Cell Index (CI) value using the xCELLigence software (version 1.2.1) by employing the equation below:

$$\mathrm{CI}({\rm{t}})=\frac{{\rm{R}}({f}_{{\rm{n}}},{\rm{t}})-{\rm{R}}({f}_{{\rm{n}}},{\rm{t}}0)}{{{\rm{Z}}}_{{\rm{n}}}}$$

CI = Cell Index

t = a given time-point

ƒ_n_ = the frequency at which the impedance measurement is carried out

R = measured impedance

Z_n_ = corresponding frequency factor of ƒn

The CI curve is plotted in real time with the mean (±SD) of at least three individual wells per treatment. All wells were coated with 1 µg/cm^2^ rat collagen diluted in 0.02 M glacial acetic acid solution (Nunc, Thermo Fisher Scientific. Cat#: 142475) and cells were seeded in 100 µl media, at a density of 60,000 cm^−2^ per well. Cells were treated when confluent, (as shown by a plateau in CI) with TLR ligands at final concentrations as detailed in the corresponding figure legends. Experiments were repeated at least 3 times.

### Analysis of Cytokine Secretion

Cells were plated at a density of 60,000 cm^−2^ onto a 24 well plate coated with 1 µg/cm^2^ rat collagen diluted in 0.02 M glacial acetic acid. Once cells had reached confluence the media was removed and replaced with the treatments as detailed in corresponding figure legends. At the appropriate time points, 100 µl of conditioned media was removed, spun down at 300 × g to remove debris and then frozen in 40 µl aliquots. Multiplexed cytokine analysis was conducted using the Cytometric Bead Array (CBA) technology (BD USA). CBAs were conducted as described previously and assayed on an Accuri C6 flow cytometer^22^. The FCAP Array software version 3.0 (BD Bioscience) was used to create standard curves for each cytokine measured, converting the mean fluorescent intensity (MFI) for each sample into a concentration. Experiments were repeated at least 2 times.

### Characterization of changes in barrier strength using ECIS ZΘ TEER technology

hCMVEC barrier strength was analysed using ECIS *ZΘ* TEER technology; a 96W20idf plate was stabilized with 10 mM cysteine for 10 minutes before collagen coating with 1 µg/cm^2^ rat collagen diluted in 0.02 M glacial acetic acid solution. Cells were seeded in 100 µl media at a density of 60,000 cm^−2^ with at least 3 individual wells measured per treatment. Confluent cells were treated at approximately 48 hours post-seeding with TLR ligands at final concentrations as detailed in the corresponding figure legends. The impedance of the hCMVEC monolayer was measured at multiple frequencies, allowing different aspects of the barrier; the Rb (resistance between the cells) and α (resistance underneath the cell), to be modelled using ECIS Software V.1.2.163.0. PC. Experiments were repeated at least 3 times.

### RT-PCR of TLR expression

For PCR analysis total RNA was isolated from untreated hCMVECs using TRIzol Reagent (Life Technologies Cat# 15596-026) as per the manufacturer’s instructions. cDNA synthesis was performed using Roche transcriptor (Roche Life Science; #03531295001) as per manufacturer’s instructions, with RNA input of 1 µg per reaction. To test functionality and determine the annealing temperature of the primers, total RNA was taken from peripheral blood mononuclear cells (PBMCs) and transcribed into cDNA using the methods above. Human blood was obtained from healthy individuals by a trained phlebotomist under institution ethical approval (ethics number 014035) held by Dr Graham. PBMC were isolated by routine density gradient leukocyte isolation method as described previously^32^.

The amplification was performed on a Kyratech Supercycler Trinity (Kyratech) using the following conditions; 95 °C for two minutes, then 35 cycles of 95 °C for 20 seconds, 55–66 °C (depending on the primer, see Table 1) for 30 seconds, and 72 °C for 30 seconds (if the product size was under 600 bp) or 60 seconds (if the product size was over 600 bp) followed by a single post-cycle extension of 72 °C for 5 minutes. PCR products were electrophoretically separated on 1% agarose gels and visualized by RedSafe nucleic acid staining (Ngaio Diagnostics). Experiments were repeated at least 3 times.

**Table 1:** Human TLR Primers designed for RT-PCR.

Name	Amplicon size (bp)	Forward 5′ to 3′	Reverse 5′ to 3′
Human GAPDH	437	CATCATCTCTGCCCCCTCTG	CCTGCTTCACCACCTTCTTG
Human TLR3	752	CTGGAAACACGCAAACCCTG	CCGCCTCAAAGTCCCTTTCT
Human TLR4	452	AAAATCCCCGACAACCTCCC	ACCCGCAAGTCTGTGCAATA
Human TLR7	579	AGCGTCCTTTCACAGACTGG	TTTTTACACGGCGCACAAGG
Human TLR8	919	GCTGACCTGCATTTTCCTGC	CAGCACCTTCAGATGAGGCA
Human TLR9	751	TGCCCAAACTGGAAGTCCTC	GAGGCCCACAGGTTCTCAAA

### Statistics

Data produced following CBA analysis, xCELLigence RTCA and ECIS experiments were subjected to one-way ANOVA statistical analysis followed by Uncorrected Fisher’s LSD using GraphPad Prism 6 to determine the experimental means and standard deviations (SD). Significance was set to p < 0.05.

## Electronic supplementary material

Supplementary figures

## References

[1] Bazzoni G, Dejana E (2004). Endothelial cell-to-cell junctions: molecular organization and role in vascular homeostasis. Physiol. Rev..

[2] Lok J (2007). Cell–cell signaling in the neurovascular unit. Neurochem. Res..

[3] Medzhitov R, Preston-Hurlburt P, Janeway CA (1997). A human homologue of the Drosophila Toll protein signals activation of adaptive immunity. Nature.

[4] Kawai T, Akira S (2010). The role of pattern-recognition receptors in innate immunity: update on Toll-like receptors. Nat. Immunol..

[5] Miyake, Y. & Yamasaki, S. In *Self and Nonself* 144–152 (Springer, 2012).

[6] Jin MS, Lee J-O (2008). Structures of the toll-like receptor family and its ligand complexes. Immunity.

[7] Janeway CA, Medzhitov R (2002). Innate immune recognition. Annu. Rev. Immunol..

[8] Barbalat R, Lau L, Locksley RM, Barton GM (2009). Toll-like receptor 2 on inflammatory monocytes induces type I interferon in response to viral but not bacterial ligands. Nat. Immunol..

[9] Faure E (2000). Bacterial lipopolysaccharide activates NF-kappaB through toll-like receptor 4 (TLR-4) in cultured human dermal endothelial cells. Differential expression of TLR-4 and TLR-2 in endothelial cells. The Journal of biological chemistry.

[10] Faure ET (2001). Bacterial lipopolysaccharide and IFN- gamma induce Toll-like receptor 2 and Toll-like receptor 4 expression in human endothelial cells: Role of NF-kappa B activation. Journal Of Immunology; J.Immunol..

[11] Nagyőszi P (2010). Expression and regulation of toll-like receptors in cerebral endothelial cells. Neurochem. Int..

[12] Li J (2013). Immune activation of human brain microvascular endothelial cells inhibits HIV replication in macrophages. Blood.

[13] Tang, S. C. *et al*. Pivotal role for neuronal Toll-like receptors in ischemic brain injury and functional deficits. *Proc. Natl. Acad. Sci. USA* **104**, 13798–13803, doi:0702553104 [pii] (2007). 10.1073/pnas.0702553104 PMC1959462 17693552

[14] Ziegler G (2007). TLR2 has a detrimental role in mouse transient focal cerebral ischemia. Biochem. Biophys. Res. Commun..

[15] Brea D (2011). Toll-like receptors 2 and 4 in ischemic stroke: outcome and therapeutic values. J. Cereb. Blood Flow Metab..

[16] Cao C-x (2007). Reduced cerebral ischemia-reperfusion injury in Toll-like receptor 4 deficient mice. Biochem. Biophys. Res. Commun..

[17] Kho D (2015). Application of xCELLigence RTCA biosensor technology for revealing the profile and window of drug responsiveness in real time. Biosensors.

[18] van Kralingen, C., Kho, D., Costa, J., Angel, C. E. & Graham, E. Exposure to Inflammatory Cytokines IL-1 beta and TNF alpha Induces Compromise and Death of Astrocytes; Implications for Chronic Neuroinflammation. *Plos One; PLoS One* **8**, 10.1371/journal.pone.0084269 (2013). 10.1371/journal.pone.0084269 PMC3868583 24367648

[19] Kho DT (2017). ECIS technology reveals that monocytes isolated by CD14+ ve selection mediate greater loss of BBB integrity than untouched monocytes, which occurs to a greater extent with IL-1β activated endothelium in comparison to TNFα. PLoS One.

[20] Kho DT, Johnson RH, O’Carroll SJ, Angel CE, Graham ES (2017). Biosensor Technology Reveals the Disruption of the Endothelial Barrier Function and the Subsequent Death of Blood Brain Barrier Endothelial Cells to Sodium Azide and Its Gaseous Products. Biosensors.

[21] Giaever I, Keese CR (1991). Micromotion of mammalian cells measured electrically. Proceedings of the National Academy of Sciences.

[22] O’Carroll SJ (2015). Pro-inflammatory TNFalpha and IL-1beta differentially regulate the inflammatory phenotype of brain microvascular endothelial cells. J. Neuroinflammation.

[23] Szeimies R-M (2004). Imiquimod 5% cream for the treatment of actinic keratosis: results from a phase III, randomized, double-blind, vehicle-controlled, clinical trial with histology. J. Am. Acad. Dermatol..

[24] Schulze HJ (2005). Imiquimod 5% cream for the treatment of superficial basal cell carcinoma: Results from a randomized vehicle‐controlled phase III study in Europe. Br. J. Dermatol..

[25] Sohn K-C (2014). Imiquimod induces apoptosis of squamous cell carcinoma (SCC) cells via regulation of A20. PLoS One.

[26] Moodley K, Angel CE, Glass M, Graham ES (2011). Real-time profiling of NK cell killing of human astrocytes using xCELLigence technology. J. Neurosci. Methods.

[27] Schoenemeyer, A. *et al*. The interferon regulatory factor, IRF5, is a central mediator of toll-like receptor 7 signaling. *The Journal of biological chemistry* **280**, 17005-17012, doi:M412584200 [pii] (2005). 10.1074/jbc.M412584200 15695821

[28] Crawford A, Angelosanto JM, Nadwodny KL, Blackburn SD, Wherry EJ (2011). A role for the chemokine RANTES in regulating CD8 T cell responses during chronic viral infection. PLoS Pathog..

[29] Lever AR (2015). Comprehensive evaluation of poly (I: C) induced inflammatory response in an airway epithelial model. Physiological reports.

[30] Szulcek, R., Bogaard, H. J. & van Nieuw Amerongen, G. P. Electric cell-substrate impedance sensing for the quantification of endothelial proliferation, barrier function, and motility. *Journal of visualized experiments: JoVE* **(85)**, 10.3791/51300 (2014). 10.3791/51300 PMC4159052 24747269

[31] Ramirez SH (2012). Activation of cannabinoid receptor 2 attenuates leukocyte–endothelial cell interactions and blood–brain barrier dysfunction under inflammatory conditions. J. Neurosci..

[32] Grimsey NL, Moodley KS, Glass M, Graham ES (2012). Sensitive and accurate quantification of human leukocyte migration using high-content Discovery-1 imaging system and ATPlite assay. J Biomol Screen.

