# Supplementary material for: The functional and inflammatory response of brain endothelial cells to Toll-Like Receptor agonists
Source: Sci Rep. 2018 Jul 4;8:10102. doi: 10.1038/s41598-018-28518-3 (PMC6031625; doi:10.1038/s41598-018-28518-3)
Supplement: Supplementary file 1 — Supplementary figures [file 41598_2018_28518_MOESM1_ESM.docx]

The functional and inflammatory response of brain endothelial cells to Toll-Like Receptor agonists.

Rebecca H Johnson^1,2^, Dan T Kho^1,2^, Simon J O’Carroll^1,3^, Catherine E Angel^4^, E Scott Graham^1,2, *^.

1 Centre for Brain Research,
2 Department of Pharmacology and Clinical Pharmacology,
3 Department of Anatomy and Medical Imaging, School of Medical Sciences, Faculty of Medical and Health Sciences, University of Auckland.

4 School of Biological Sciences, Faculty of Science, University of Auckland.

Corresponding author; Dr E Scott Graham, Centre for Brain Research, Department of Pharmacology and Clinical Pharmacology, School of Medical Sciences, Faculty of Medical and Health Sciences, University of Auckland. [s.graham@auckland.ac.nz](mailto:s.graham@auckland.ac.nz)

**Supplemental Figure 1;** Analysis of hCMVEC responsiveness to TLR ligands measured using xCELLigence RTCA Biosensor technology. TLR agonists were administered to confluent hCMVECs 48 hours post-seeding on an xCELLigence 96 well plate with (a) Pam2CSK4, (b) Pam3CSK4, (c) R-848, and (d) CpG-ODN 2006. Each ligand is administered at 5 µg/ml (red), 500 ng/ml (green), and 50 ng/ml (blue) with media-only control (black). Curves represent the mean ± SD of three individual wells, and are representative of at least three independent experiments.

**Supplemental Figure 2**; Raw data of hCMVEC responsiveness to TLR ligands measured using xCELLigence RTCA Biosensor technology. TLR agonists were administered to confluent hCMVECs 48 hours post-seeding on an xCELLigence 96 well plate with Poly(I:C) 5 µg/mL (blue), Imiquimod 5 µg/mL (green), LPS 100 ng/ml (red), or media-only control (black). Curves represent the mean ± SD of three individual wells with a red arrow indicating ligand addition and are representative of at least three independent experiments.


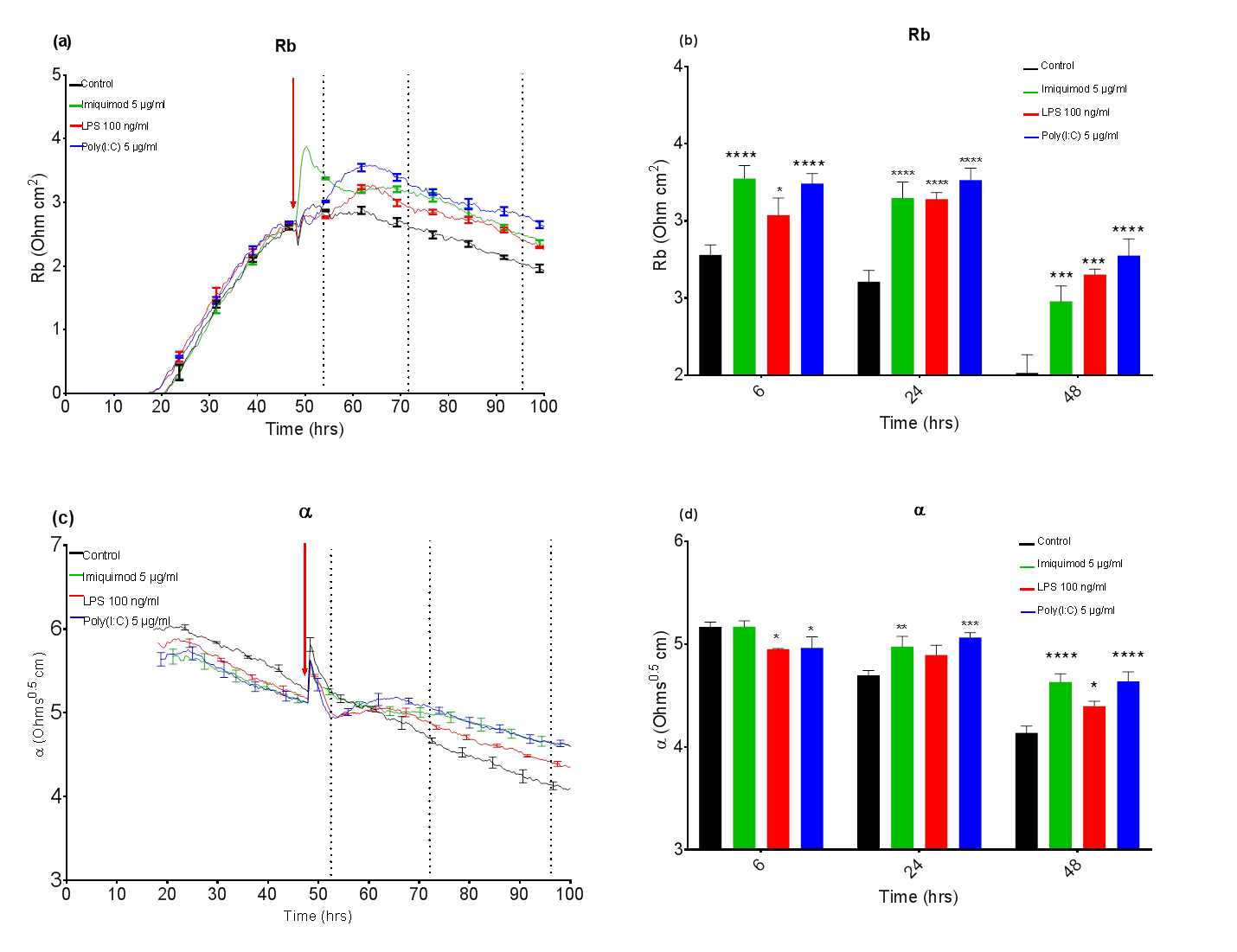


**Supplemental Figure 3**; Modelled Rb and α of hCMVEC adhesion. TLR agonists were administered to confluent hCMVECs 48 hours post-seeding (represented by red arrow) on an ECIS 96W20idf plate with LPS (100 ng/ml) red, Imiquimod (5 µg/mL) green, Poly(I:C) (5 µg/mL) blue, and untreated media-only control, black. Multi-frequency measurements were taken and this data was later modelled using ECIS Software V.1.2.163.0 PC. (a) Rb, with a red arrow indicating ligand addition and dashed lines indicating 4, 24 and 48 hours following ligand addition. (b) Rb of hCMVEC 4, 24 or 48 hours following addition of Poly(I:C), LPS, Imiquimod or media-only control. (c) α, with a red arrow indicating ligand addition and dashed lines indicating 4, 24 and 48 hours following ligand addition. (d) α of hCMVEC 4, 24 or 48 hours following addition of Poly(I:C), LPS, Imiquimod or media-only control. The modelled data is from a single experiment where each curve represents the mean ± SD of three individual wells and are representative of at least three independent experiments.

**Supplemental Figure 4**; Modelled Rb of hCMVEC adhesion. TLR agonists were administered to confluent hCMVECs 48 hours post-seeding (represented by red arrow) on an ECIS 96W20idf plate with **(a)** LPS (100 ng/ml), Imiquimod (5 µg/mL), LPS (100 ng/ml) plus Imiquimod (5 µg/mL), or untreated media-only control **(b)** Poly(I:C) (5 µg/mL), Imiquimod (5 µg/mL), Poly(I:C) (5 µg/mL) plus Imiquimod (5 µg/mL), ,and untreated media-only control. Multi-frequency measurements were taken and this data was later modelled using ECIS Software V.1.2.163.0 PC. The modelled data is from a single experiment where each curve represents the mean ± SD of three individual wells and are representative of at least three independent experiments.

**Supplemental Figure 5;** Analysis of hCMVEC responsiveness to structurally different TLR7 ligands measured using xCELLigence RTCA Biosensor technology. TLR7 agonists were administered to confluent hCMVECs 48 hours post-seeding on an xCELLigence 96 well plate with (a) Loxoribine, (b) CL307, (c) Gardiquimod, and (d) CL097. Each ligand is administered at 5 µg/ml (green) and 1 µg/ml (blue) with media-only control (black). Curves represent the mean ± SD of three individual wells, and are representative of at least three independent experiments.


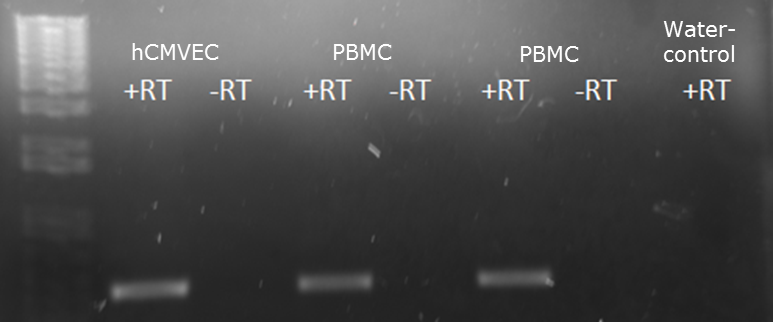


**Supplemental Figure 6;** GAPDH expression in hCMVEC and PBMC cDNA. RT-PCR of untreated hCMVEC cDNA for GAPDH. PCR products were separated by agarose gel electrophoresis and visualized using RedSafe. A 100 bp DNA ladder was used for amplicon sizing.
